# Functional Promoter -308G>A Variant in Tumor Necrosis Factor α Gene Is Associated with Risk and Progression of Gastric Cancer in a Chinese Population

**DOI:** 10.1371/journal.pone.0050856

**Published:** 2013-01-10

**Authors:** Yan Hong, Zhijun Ge, Changrui Jing, Jun Shi, Xiao Dong, Fengying Zhou, Meilin Wang, Zhengdong Zhang, Weida Gong

**Affiliations:** 1 Department of Surgery, Yixing People's Hospital, Yixing, China; 2 Department of Plastic Surgery, Wuxi No.2 People's Hospital, Wuxi, China; 3 Department of Surgery, Yixing Cancer Hospital, Yixing, China; 4 Department of Breast Surgery, Wuxi Maternal and Child Health Hospital, Wuxi, China; 5 Department of Molecular and Genetic Toxicology, School of Public Health, Nanjing Medical University, Nanjing, China; 6 Cancer Center, Nanjing Medical University, Nanjing, China; University of Porto, Portugal

## Abstract

**Background:**

Tumor necrosis factor-α (TNF-α) plays a crucial role in the development and progression of gastric cancer. A functional polymorphism, -308 G>A (rs1800629), which is located in the promoter of *TNFA* gene, has been suggested to alter the production of TNF-α and influence cancer risk. In the present study, we sought to investigate whether this polymorphism has effects on the risk and progression of gastric cancer in a Chinese population.

**Methods:**

We genotyped the *TNFA* -308 G>A polymorphism using the TaqMan method in a two-stage case-control study comprising a total of 1686 gastric cancer patients and 1895 cancer-free subjects. The logistic regression was used to assess the genetic associations with occurrence and progression of gastric cancer.

**Results:**

We found a significant association between the variant genotypes and increased risk of gastric cancer [*P* = 0.034, odds ratio (OR) = 1.39, 95% confidence interval (CI) = 1.01–1.67, GA/AA vs. GG]. Similar results were observed in the follow-up replication study. When combined the data from the two studies, we found a more significant association (*P* = 0.001, OR = 1.34, 95%CI = 1.13–1.59), especially for older subjects (>65 years). Furthermore, the patients carrying the variant genotypes had a significantly greater prevalence of T4 stage of disease (*P* = 0.001, OR = 2.19, 95%CI = 1.39–3.47) and distant metastasis (*P* = 0.013, OR = 1.61, 95%CI = 1.10–2.35).

**Conclusions:**

Our results suggest that the functional promoter -308 G>A polymorphism in *TNFA* influence the susceptibility and progression of gastric cancer in the Chinese population.

## Introduction

Gastric cancer is the fourth most common cancer and the second leading cause of cancer-related death worldwide, and particularly prevalent in certain countries, including China [1], [2]. Although the exact etiology of gastric cancer remains to be identified, accumulating epidemiological studies have shown that diet, smoking, alcohol and especially *Helicobacter pylori* (*H. pylori*) infection, are well-known causes of gastric cancer [3], [4]. However, a high prevalence of these risk factors do not always result in a high incidence of gastric cancer, which suggests that other susceptible factors such as genetic variations and environmental differences may additionally contribute to gastric carcinogenesis. Recently, emerging evidence has suggested that genetic polymorphisms in candidate genes were associated with susceptibility to gastric cancer [5], [6], [7].

It has been suggested that chronic inflammation in gastric mucosa induced by the *H. pylori* infection is a critical step in the development of gastric cancer [8], [9]. In that case, genetic variations in inflammation-related cytokine genes may be potential susceptibility factors for this disease. One of the important cytokines associated with *H. pylori* infection is the tumor necrosis factor (TNF) –α which is encoded by the *TNFA* gene [10]. TNF-α is a pleiotropic cytokine mainly produced by activated monocytes and macrophages and plays an important role in the inflammatory response [11]. It has been suggested that over expression of TNF-α showed a significant severity-dose-response as risk markers from pre-neoplastic lesions to gastric cancer [12].

The *TNFA* gene is located within the human leukocyte antigen class III region on chromosome 6 (6p21) and is highly polymorphic. Polymorphisms in the *TNFA* gene have been shown to greatly influence its expression level [13]. Therefore, many studies attempted to investigate whether polymorphisms in the promoter of *TNFA* could be used as putative determinant factor of susceptibility for various diseases, including gastric cancer. Among the many polymorphisms in *TNFA* promoter, the *TNFA*-308 G>A polymorphism were wildly studied and there is functional study suggesting that the -308A allele is associated with increased TNF-α production [14]. As for gastric cancer, a number of studies were conducted to investigate the associations between the *TNFA*-308 G>A polymorphism and gastric cancer in various populations, however, the results form these studies remain inclusive [15], [16], [17], [18], [19]. To date, two published meta-analyses on this issue have suggested that the -308 G>A polymorphism was associated with gastric cancer in Western populations, but not in Asian populations [20], [21]. Herein, in the present study, we conducted a relative large two-stage case-control study that included a total of 1686 gastric cancer patients and 1895 cancer-free subjects to further evaluate the influence of the *TNFA* -308 G>A polymorphism on gastric cancer risk and progression in a Chinese population.

## Materials and Methods

### Ethics statement

The study was approved by the Institutional Review Board of the Nanjing Medical University, Nanjing, China. At recruitment, written informed consent was obtained from all participants involved in this study.

### Study population

This is an ongoing molecular epidemiologic study of gastric cancer conducted in the First Affiliated Hospital of Nanjing Medical University, Nanjing, China. The design of the study and the inclusion criteria of the subjects were previously described elsewhere [22]. In brief, two independent hospital-based case-control studies were included in the present study. Overall, the test set included 750 gastric cases and 835 age and sex-matched controls recruited at the second affiliated hospital of Nanjing Medical University, Nanjing and Cancer Hospital of Nantong City, Nantong, China from March, 2006 to January, 2010, and the validation set included 936 cases and 1,060 controls enrolled from Yixing People's Hospital, Yixing, China from January, 1999 to December, 2006. All subjects were ethnic Han Chinese coming from different families and had no blood relationship. All the patients were newly diagnosed with histopathologically confirmed, incident gastric cancer and were consecutively recruited without restriction of age and sex. Histological sections of all cases were reviewed by two pathologists independently. Those patients that had previous cancer, metastasized cancer from other or unknown origin, or previous radiotherapy or chemotherapy were excluded. The controls were recruited from healthy subjects who were seeking physical examination in the outpatient departments at the corresponding hospital and were frequency-matched to the cases by age (±5 years) and sex. The cancer-free controls were genetically unrelated to the cases and had no individual history of cancer. Each patient donated 5 ml venous blood after providing a written informed consent. The response rate for case and control subjects was both above 85%.

### DNA extraction and genotyping

The whole genomic DNA was isolated and purified from leucocytes of peripheral blood by proteinase K digestion and phenol/chloroform extraction. The *TNFA* -308 G>A polymorphism was genotyped using the TaqMan-MGB method (Applied Biosystems, Foster City, CA, USA), which uses two allele-specific TaqMan MGB probes and a PCR primer pair to detect the specific SNP target. The sequence of the primers and probes are available on request. The reaction mixture of 10 µL contained 20 ng genomic DNA, 3.5 µL of 2× TaqMan Genotyping Master Mix, 0.25 µL of the primers and probes mix and 6.25 µL of double distilled water. The amplification was performed under the following conditions: 50°C for 2 min, 95°C for 10 min followed by 45 cycles of 95°C for 15 sec, and 60°C for 1 min. Following the manufacturer's instructions, amplifications were conducted in the 384-well ABI 7900HT Real Time PCR System (Applied Biosystems, Foster City, CA, USA) and the allelic discrimination were performed using the SDS 2.4 software (Applied Biosystems, Foster City, CA, USA). The genotyping rates of these SNPs were all above 98%. To ensure the accuracy of genotyping, two negative experimental control (water) and two positive experimental controls with known genotype were included in each reaction plate. In addition, about 5% of the samples were randomly selected for repeated genotyping for confirmation; and the results were 100% concordant.

### Statistical analysis

Before further analysis, the allele frequencies of *TNFA* -308G>A polymorphism in the controls of test set, validation set and combined set were assessed against departure from Hardy-Weinberg equilibrium (HWE) using a goodness-of-fit χ2-test. Differences in the distributions of age, sex and frequencies of genotypes of the *TNFA* -308G>A polymorphism between the cases and controls were evaluated by Pearson's χ2 test. The associations between the -308G>A genotypes and risk of gastric cancer as well as the clinical characteristics of the patients were measured by computing odds ratios (ORs) and 95% confidence intervals (CIs) from unconditional logistic regression analysis with the adjustment for age and sex. All the statistical analyses were performed with the software SAS 9.1.3 (SAS Institute, Cary, NC, USA) and a two-side *P* value of less than 0.05 was considered as statistically significant.

## Results

### Characteristics of the study population

The frequency distributions of demographic characteristics and clinical features of the two sets of study group are shown in Table 1. The cases and controls were matched by age and sex (*P* = 0.383 and 0.424 in the test set and *P* = 0.164 and 0.061 in the validation set, respectively). Although more older subjects (>65years) and female subjects were presented in the test set than in the validation set, no significant difference in age and sex between the cases and controls was observed when we combined the two populations (Table 1). In the test set, there were 295 cardia gastric cancer (CGC) patients and 455 non-cardia gastric cancer (NCGC) patients; 406 patients diagnosed as diffuse type of gastric cancer and 299 as intestinal type. Clinical TNM stage is categorized according to depth of invasion, lymph node metastasis and distant metastasis. The percent of TNM stage of patients in the test set from I to IV were 26.8%, 22.0%, 35.5%, and 15.7%, respectively. Of the 936 patients in the validation set, 358 were CGC and 578 were NCGC; 539 patients diagnosed as diffuse type of gastric cancer and 397 as intestinal type. Approximately 27.7%, 19.8%, 42.2% and 10.3% of the patients were found to have TNM stage I, II, III and IV diseases, respectively.

**Table 1:** Patient characteristics and clinical features.

Variables	Test set (N, %)		*P* *	Validation set (N, %)		*P* *	Combined (N, %)		*P* *
	Cases	Controls		Cases	Controls		Cases	Controls	
Age (years)									
≤65	430 (57.3%)	460 (55.2%)	0.383	601 (64.2%)	712 (67.2%)	0.164	1031(61.2%)	1172(61.9%)	0.655
>65	320 (42.7%)	374 (44.8%)		335 (35.8%)	348 (32.8%)		655 (38.8%)	722 (38.1%)	
Sex									
Male	510 (68.0%)	551 (66.1%)	0.414	721 (77.0%)	778 (73.4%)	0.061	1231(73.0%)	1329(70.2%)	0.060
Female	240 (32.0%)	283 (33.9%)		215 (23.0%)	282 (26.6%)		455 (27.0%)	565 (29.8%)	
Site									
Cardia	295 (39.3%)			358 (38.3%)			653 (38.7%)		
Non-cardia	455 (60.7%)			578 (61.7%)			1033(61.3%)		
Histological types									
Diffuse	406 (57.6%)			539 (57.6%)			945 (57.6%)		
Intestinal	299 (42.4%)			397 (42.4%)			696 (42.4%)		
Depth of invasion									
T1	120 (17.4%)			147 (15.7%)			267 (16.4%)		
T2	120 (17.4%)			199 (21.3%)			319 (19.6%)		
T3	350 (50.7%)			543 (58.0%)			893 (55.0%)		
T4	101 (14.5%)			45 (5%)			146 (9.0%)		
Lymph node metastasis									
Positive	333 (44.4%)			371 (39.6%)			704 (41.8%)		
Negative	417 (55.6%)			565 (60.4%)			982 (58.2%)		
Distant metastasis									
M0	616 (87.3%)			878 (93.8%)			1494(91.0%)		
M1	90 (12.7%)			58 (6.2%)			148 (9.0%)		
TNM stage									
I	189 (26.8%)			259 (27.7%)			448 (27.3%)		
II	155 (22.0%)			185 (19.8%)			340 (20.7%)		
III	250 (35.5%)			395 (42.2%)			645 (39.3%)		
IV	111 (15.7%)			97 (10.3%)			208 (12.7%)		

* Two-sided χ2 test.

### Association between the *TNFA* -308 G>A polymorphism and gastric cancer risk

The genotyping results of the *TNFA* -308 G>A polymorphism among the patients and controls in the two study set are presented in the Table 2. The observed genotype frequencies in the controls of test set, validation set and the combined set were all conformed to the HWE (*P* = 0.481, 0.448 and 0.946, respectively). We then used Pearson's χ2 test and logistic regression analysis to assess the association between *TNFA* -308 G>A polymorphism and gastric cancer risk. In the test set, we found that there were more variant genotypes (GA/AA) in the case group than that in the control group (*P* = 0.034). Compared with the individuals with GG genotype, we found those subjects with GA/AA genotypes had an increased risk of gastric cancer (adjusted OR = 1.39, 95%CI = 1.01–1.67). Similar associations were observed in the replication study (GA vs. GG: OR = 1.29, 95%CI = 1.02–1.65 and GA/AA vs. GG: OR = 1.31, 95%CI = 1.03–1.65). When we combined data from the two studies, a more significant association was observed (GA vs. GG: OR = 1.33, 95%CI = 1.12–1.59 and GA/AA vs. GG: OR = 1.34, 95CI% = 1.13–1.59).

**Table 2:** Genotype and allele frequencies of *TNF-α* -308 G>A polymorphism among the cases and controls and their associations with risk of gastric cancer.

Genotypes	Cases, N (%)	Controls, N (%)	*P* *	OR (95% CI)*	Adjusted OR (95% CI)†
The test set					
GG	589 (78.5)	690 (82.7)	0.098	1.00 (reference)	1.00 (reference)
GA	154 (20.6)	139 (16.7)		1.30 (1.01–1.67)	1.29 (1.00–1.66)
AA	7 (0.9)	5 (0.6)		1.64 (0.52–5.19)	1.59 (0.50–5.06)
GA+AA	161 (21.5)	144 (17.3)	0.034	1.31 (1.02–1.68)	1.39 (1.01–1.67)
A allele	168 (11.2)	149 (8.9)	0.034	1.29 (1.02–1.62)	-
The validation set	1332	1519			
GG	746 (79.7)	895 (84.4)	0.022	1.00 (reference)	1.00 (reference)
GA	179 (19.1)	156 (14.7)		1.37 (1.09–1.74)	1.29 (1.02–1.65)
AA	11 (1.2)	9 (0.9)		1.46 (0.60–3.55)	1.44 (0.59–3.54)
GA+AA	190 (20.3)	165 (15.6)	0.002	1.38 (1.10–1.74)	1.31 (1.03–1.65)
A allele	201 (10.7)	174 (0.2)	0.006	1.35 (1.09–1.54)	-
Combined	1671	1946			
GG	1335 (79.2)	1585 (83.7)	0.002	1.00 (reference)	1.00 (reference)
GA	333 (19.8)	295 (15.6)		1.34 (1.13–1.59)	1.33 (1.12–1.59)
AA	18 (1.1)	14 (0.7)		1.52 (0.76–3.08)	1.54 (0.76–3.14)
GA+AA	351 (20.8)	309 (16.3)	0.001	1.35 (1.14–1.60)	1.34 (1.13–1.59)
A allele	369 (10.9)	323 (8.5)	0.001	1.32 (1.13–1.54)	-

* Two-sided χ2 test 3003 3465.

† Were adjusted for age and sex. OR, odds ratio; CI, confidence interval.

To evaluate if the *TNFA* -308 G>A polymorphism plays different role in subgroups of age, sex, tumor site and histological types, we then performed stratified analysis based on these variables. As shown in Table 3, the stratified analysis showed that the increased risk associated with the *TNFA* -308GG/GA genotypes was more evident among older subjects (>65years) (*P*<0.001, OR = 1.70, 95%CI = 1.28–2.25). Besides, no significant difference was observed in the stratification of sex, tumor site and histological types.

**Table 3:** Stratification analyses between *TNFA* -308 G>A polymorphism and gastric cancer risk in the combined group.

Variables	*TNFA* -308 G>A genotypes				*P* *	Adjusted OR (95% CI)†
	GG (N, %)		GA/AA (N, %)			
	Cases	Controls	Cases	Controls		
Age (years)						
≤65	820 (79.5)	962 (82.4)	211 (20.5)	206 (17.6)	0.091	1.17 (0.95–1.45)
>65	515 (78.6)	623 (85.8)	140 (21.4)	103 (14.2)	<0.001	1.70 (1.28–2.25)
Sex						
Male	970 (78.8)	1095 (82.4)	261 (21.2)	234 (17.6)	0.021	1.27 (1.04–1.55)
Female	365 (80.2)	490 (86.7)	90 (19.8)	75 (13.3)	0.005	1.63 (1.15–2.32)
Site						
Cardia	517 (79.2)	1585 (83.7)	136 (21.8)	309 (16.3)	0.009	1.34 (1.07–1.68)
Non-cardia	818 (79.2)	1585 (83.7)	215 (21.8)	309 (16.3)	0.002	1.35 (1.12–1.64)
Histological types						
Diffuse	753 (79.7)	1585 (83.7)	192 (20.3)	309 (16.3)	0.008	1.32 (1.08–1.61)
Intestinal	551 (29.2)	1585 (83.7)	145 (20.8)	309 (16.3)	0.007	1.34 (1.08–1.67)

* Two-sided χ2 test.

† Were adjusted for age, sex. OR, odds ratio; CI, confidence interval.

### Association between the *TNFA* -308 G>A polymorphism and clinical characteristics of the patients with gastric cancer

Polymorphisms in candidate genes have been suggested to influence cancer progression. We then evaluated whether the *TNFA* -308 G>A polymorphism has effects on the clinical characteristics of the patients, including TNM stage, distant metastasis, lymph node metastasis and depth of tumor infiltration. As shown in Table 4, we found that the patients carrying the variant genotypes had a significantly greater prevalence of T4 stage of invaded depth (*P* = 0.001, OR = 2.19, 95%CI = 1.39–3.47) and distant metastasis (*P* = 0.013, OR = 1.61, 95%CI = 1.10–2.35), indicating the polymorphism might be associated with gastric cancer progression.

**Table 4:** The association between *TNF-α*-308 G>A polymorphism and the progression of gastric cancer.

Category	GG (N, %)	GA/AA (N, %)	*P* *	Adjusted OR (95% CI)*
Depth of invasion				
T1	216 (80.9)	51 (19.1)		1.00 (reference)
T2	253 (79.3)	66 (20.7)	0.654	1.10 (0.73–1.65)
T3	726 (81.3)	167 (18.1)	0.861	0.97 (0.68–1.37)
T4	96 (65.8)	50 (34.3)	0.001	2.19 (1.39–3.47)
Lymph node metastasis				
Positive	549 (78.0)	155 (22.0)		1.00 (reference)
Negative	786 (80.0)	196 (20.0)	0.305	0.88 (0.70–1.12)
Distant metastasis				
M0	1199 (80.3)	295 (19.8)		1.00 (reference)
M1	106 (71.6)	42 (28.4)	0.013	1.61 (1.10–2.35)
TNM stage				
I	355 (79.2)	93 (20.8)		1.00 (reference)
II	268 (78.8)	72 (21.2)	0.915	1.02 (0.72–1.44)
III	530 (82.2)	115 (17.8)	0.220	0.83 (0.61–1.12)
IV	151 (72.6)	57 (27.4)	0.061	1.44 (0.98–2.11)

* were adjusted for age and sex in logistic regression model. CI: confidence interval; OR: odds ratio.

## Discussion

The main findings of our study are the significant associations between the *TNFA* -308 G>A polymorphism and gastric cancer risk and progression. TNF-α is an important immune mediator in inflammatory response and has been suggested to be an endogenous tumor promoter in human carcinogenesis [23], [24], [25]. In gastric cancer, Senthilkumar *et al.* demonstrated that TNF-α mRNA and protein expressions were significantly increased in gastric adenocarcinoma patients, by using RT-PCR, western blotting and immunohistochemistry [12]. The influence of the -308G>A polymorphism on the expression level of *TNFA* has been previously investigated. Karen *et al.* showed that the -308G>A polymorphism could affect the affinity of factor binding and result in a factor binding to the -308A allele but not the -308G allele, which then lead to an increased expression of *TNFA* [14]. Considering that TNF-α is up-regulated in gastric cancer, it is possible that individuals carrying the -308A allele (ie. GA/AA), which is associated with increased level of TNF-α, have an increased risk for gastric cancer. Therefore, it is biologically plausible that the *TNFA* -308G>A confers individuals' susceptibility to gastric cancer.

To date, several molecular epidemiological studies have been conducted to investigate the association between the *TNFA* -308G>A polymorphism and gastric cancer risk. However, the results from these studies remain inconclusive. There are two published meta-analyses on the associations between the *TNFA* -308G>A and gastric cancer risk that have identified this polymorphism as a risk factor for gastric cancer in Caucasian populations but not in Asian populations [20], [21]. When reviewed the two meta-analyses, we found only 3 studies with a total of 513 cases and 714 controls and 5 studies with a total of 833 cases and 1375 controls in Chinese populations were included in the meta-analyses of Zhang *et al.* [20] and Gorouhi *et al.* [21], respectively (Table 5). The number of studies and the sample size of these studies may be too limited to generate a stable result. Of these studies, only Lu *et al.* found a significant association between *TNFA* -308GA genotype and increased risk of gastric cancer risk (OR = 1.81, 95%CI = 1.04–3.14). Our study further identify the *TNFA* -308G>A polymorphism as a risk factor for gastric cancer in Chinese population, which may be of value in evaluating the role of TNF- α in gastric carcinogenesis. As indicated by Power and Sample Size Calculation software, our study had a 96% power to detect an OR of 1.50 or greater and a minimal OR of 0.62 with an exposure frequency of 9% at the current sample size (1686 cases and 1895 controls), which suggests that the sample size of our study is efficient.

**Table 5:** Comparison of the sample size of the studies conducted in Chinese populations.

Studies	Meta-analysis 1*		Meta-analysis 2†		Our study	
	Case	Control	Case	Control	Case	Control
Li *et al.* [15]	59	264	59	264	-	-
Lu *et al.* [16]	250	300	250	300	-	-
Wu *et al.* [17]	204	210	204	210	-	-
Fei *et al.* [18]	56	164	-	-	-	-
Guo *et al.* [19]	264	437	-	-	-	-
Our study	-	-	-	-	1686	1895
Total	833	1375	513	714	1686	1895

* The mete-analysis of Gorouhi *et al* [21].

† The mete-analysis of Zhang *et al* [20].

In addition, we found that the *TNFA* -308G>A polymorphism was associated with increased risk of gastric cancer among subgroups of older subjects (age>65 years) rather than younger subjects. This observation is well supported by a large body of studies, which link weak immune system with ageing [26], [27]. The immune system of aged individuals undergoes alterations that may account for an increased susceptibility to malignancies [26]. It has been proposed that high expression of TNF- α might be involved in tumor growth and spread through influencing tissue remodeling and stromal development [28], [29]. In our study, we also found the *TNFA* -308G>A polymorphism was associated with clinical aggressiveness such as advanced tumor stage and distant metastasis. Similar observation has also been reported in other study [30]. If confirmed by additional studies, this polymorphism might help to accurately predict the clinical course of gastric cancer. However, these results should be interpreted with cautious because there is possibility that the associations with poor prognosis are due to a late stage at diagnosis.

When interpreting our results, some other limitations should also be concerned. First, since our study was hospital-based design, the possibility of selection bias of subjects that were associated with a particular genotype might exist. However, the genotype distributions in our controls were similar to distributions reported in other Chinese studies and conformed to HWE. Therefore, the selection bias in terms of genotype distributions would not be substantial. Second, *H. pylori* infection status is a well-known cause of gastric cancer and has been suggested to influence the expression of TNF- α [31]. Genetic variations in *TNFA* may interact with this factor to affect gastric caner risk. Unfortunately, lack of available information on *H. pylori* infection status in our study limited us to further explore these interactions. Thus, further studies with more detailed data on *H. pylori* infection are warranted.

In conclusion, our results suggest the -308G>A polymorphism in the promoter of *TNFA* has a significant influence on the occurrence and progression of gastric in the Chinese population. Although the associations appeared to be statistically significant in our population, these findings should be independently verified by other prospective studies with larger sample size simultaneous of well-matched controls and unbiased-matched homogeneous participants.
